# Draft Genome Sequence of *Bacillus licheniformis* S127, Isolated from a Sheep Udder Clinical Infection

**DOI:** 10.1128/genomeA.00971-15

**Published:** 2015-10-01

**Authors:** Ievgenia Ostrov, Noa Sela, Mor Freed, Nihaya Khateb, Miriam Kott-Gutkowski, Dana Inbar, Moshe Shemesh

**Affiliations:** a Department of Food Quality and Safety, Institute of Postharvest and Food Sciences, Agricultural Research Organization, The Volcani Center, Bet-Dagan, Israel; b Department of Plant Pathology and Weed Research, Agricultural Research Organization, The Volcani Center, Bet-Dagan, Israel; c Israel Dairy Board, Yehud, Israel; d Microarray Service Laboratory, Core Research Facility at the Medical School, The Hebrew University of Jerusalem, Jerusalem, Israel

## Abstract

*Bacillus licheniformis* is a Gram-positive biofilm- and endospore-forming bacterium, which contaminates dairy products and can be pathogenic to humans. The draft genome sequencing for *B. licheniformis* strain S127 is reported here, providing genetic data relevant to the ability of this strain to sustain its survival in the dairy industry.

## GENOME ANNOUNCEMENT

*Bacillus licheniformis* is a Gram-positive biofilm- and endospore-forming organism that belongs to the *Bacillus* genus (1). *B. licheniformis* has been associated with food spoilage, a range of clinical conditions, including food poisoning in humans, and with bovine toxemia and abortions (2, 3). *B. licheniformis* is one of the predominant pathogenic *Bacillus* species found in raw milk and at all stages of dairy processing (4). The source of toxin-producing isolates of *B. licheniformis* might be cows with a history of mastitis (2, 3, 5). The ability of *B. licheniformis* to form biofilms allows it to sustain its presence in dairy plants (6, 7). Thus, bioﬁlms of *B. licheniformis* might present a serious microbiological problem in the dairy industry, which can lead to economic losses and threaten the health of consumers (8).

During the screening of several isolates of *Bacillus* species from Israeli dairy processing plants, we identified a new strain, which was characterized by the formation of a robust biofilm. In order to get insights into its biofilm-forming ability, a draft genome sequence was generated for the strain, which was designated *B. licheniformis* S127. The strain was isolated from a sheep udder clinical infection at the Ein-Harod sheep pen (Ihud, Israel). DNA extraction was performed using the GenElute bacterial genomic DNA kit (Sigma Aldrich, St. Louis, MO, USA), according to the manufacturer’s instructions. The draft genome sequence of the strain was determined by *de novo* assembly of paired-end MiSeq Illumina sequence data of 3,719,956 paired-end reads, with a length of 250 bp from each side. The DNA of *B. licheniformis* S127 was prepared for sequencing using the Nextera library preparation kit (Epicentre, Madison, WI). Assembly was done using the A5-mideq pipeline (9), generating 48 contigs, with an *N*_50_ of 459,307 bp and a median coverage of 179-fold. The genome size of *B. licheniformis* S127 is 4,559,800 bp, and its G+C content is 45.5%. Annotation was performed in RAST (10) before being submitted to the NCBI. *B. licheniformis* S127 has 4,923 predicted genes; of the 4,806 that are predicted to be protein-coding sequences, there were 81 tRNA genes and 36 ribosomal rRNA genes. The genome of *B. licheniformis* S127 highly resembles the genome of *B. licheniformis* ATCC 14580 (accession no. NC_006270) (11).

Under the conditions of a dairy plant, bacteria can initiate a developmental pathway leading to the formation of biofilms (8). Sporulation transcriptional activator (Spo0A) is a critical regulator for the entrance of bacteria into the biofilm formation pathway (12). A BLAST analysis was performed to identify sequences in the draft genome sharing high sequence similarity to Spo0A. The putative Spo0A gene in strain S127 shows 98 and 97% similarity to the sequence encoding Spo0A in two sequenced strains of *B. licheniformis*, 9945A and G, respectively. The histidine kinases C and D (KinC and KinD, respectively), which activate Spo0A by phosphorylation (12), were also found to be conserved in *B. licheniformis* S127. Thus, the KinC and KinD proteins show 100 and 99% similarity to the KinC and KinD of the *B. licheniformis* strain 9945A, respectively.

### Nucleotide sequence accession numbers.

The *B. licheniformis* S127 whole-genome shotgun project has been deposited at DDBJ/EMBL/GenBank under the accession number LFIM00000000. The version described in this paper is version LFIM01000000.
